# Impact of medium composition on cefiderocol susceptibility testing: comparison of CLSI/EUCAST-compliant and commercial broth microdilution methods

**DOI:** 10.1093/jacamr/dlag178

**Published:** 2026-08-24

**Authors:** Pia Turowski, Niels Pfennigwerth, Sören G Gatermann

**Affiliations:** Department of Medical Microbiology, German National Reference Centre for Multidrug-Resistant Gram-Negative Bacteria, Ruhr-University Bochum, Bochum, Germany; Department of Medical Microbiology, German National Reference Centre for Multidrug-Resistant Gram-Negative Bacteria, Ruhr-University Bochum, Bochum, Germany; Department of Medical Microbiology, German National Reference Centre for Multidrug-Resistant Gram-Negative Bacteria, Ruhr-University Bochum, Bochum, Germany

## Abstract

**Objectives:**

Cefiderocol is a siderophore cephalosporin active against multidrug-resistant Gram-negative bacteria. However, accurate cefiderocol MIC determination remains challenging due to methodological variability. This study investigated whether differences between a CLSI/EUCAST-compliant in-house BMD method and the commercial UMIC system influence MIC determination and susceptibility interpretation.

**Methods:**

A total of 119 clinical isolates, including 44 *Escherichia coli* and 75 *Klebsiella pneumoniae*, collected by the German National Reference Centre for Multidrug-resistant Gram-negative Bacteria between September 2022 and September 2024, were analysed. DDT inhibition zone diameters ranged from 6 to 30 mm. Cefiderocol MICs were determined using an in-house BMD reference method and the commercial UMIC system and compared according to EUCAST breakpoints.

**Results:**

For isolates with DDT zones <21 mm, in-house BMD classified 35 isolates as susceptible and 27 as resistant, whereas UMIC classified 31 as susceptible and 31 as resistant. Isolates with DDT zones within the EUCAST area of technical uncertainty (ATU, 21–23 mm) and ≥24 mm showed high categorical concordance across MIC methods. Overall categorical agreement and essential agreement between the in-house BMD method and the commercial UMIC system were 81.5% and 60.5%, respectively. In-house BMD MICs clustered at 1–4 mg/L, whereas UMIC MICs showed broader distributions. These findings coincided with differences in calcium, magnesium and zinc concentrations.

**Conclusions:**

Method-dependent variability in cefiderocol MIC determination may affect susceptibility interpretation near clinical breakpoints. Differences in testing conditions, particularly medium composition, may contribute to the observed differences. DDT showed good categorical concordance with both BMD methods for clearly susceptible isolates.

## Introduction

Cefiderocol is a siderophore cephalosporin active against multidrug-resistant Gram-negative bacteria, including carbapenem-resistant Enterobacterales.^1,2^ Because its activity depends on bacterial iron uptake under iron-limited conditions, accurate MIC determination requires iron-depleted CAMHB (ID-CAMHB).^3–5^ Minor differences in testing conditions may influence MIC values, particularly near clinical breakpoints.^6^ However, comparability between CLSI/EUCAST-compliant in-house BMD and commercial BMD systems remains insufficiently characterized.^7,8^ In this study, cefiderocol susceptibility was assessed using DDT, an in-house CLSI/EUCAST-compliant BMD method, and the commercial UMIC BMD system in clinical *Escherichia coli* and *Klebsiella pneumoniae* isolates selected to represent a broad range of DDT inhibition zone diameters. The study aimed to compare categorical agreement, evaluate differences in MIC distributions and identify methodological factors affecting susceptibility interpretation according to EUCAST breakpoints.

## Material and methods

### Bacterial isolates

A total of 119 clinical isolates, including 44 *E. coli* and 75 *K. pneumoniae*, were randomly selected from the strain collection of the German National Reference Centre (NRZ) for Multidrug-resistant Gram-negative Bacteria. Isolates with cefiderocol inhibition zone diameters of 6–30 mm were included to cover a broad susceptibility range. The isolate collection comprised both carbapenemase-producing and non-carbapenemase-producing isolates representative of the diversity of resistance mechanisms encountered in routine reference diagnostics. Species identification was performed by MALDI-TOF MS. DDT was performed according to EUCAST methodology using cefiderocol discs (30 µg).^6^ Inhibition zone diameters were measured and interpreted according to the current EUCAST clinical breakpoints, with isolates classified as susceptible (≥24 mm), resistant (<21 mm), or within the area of technical uncertainty (ATU; 21–23 mm).^9^

### Cefiderocol MIC determination

Cefiderocol MICs were determined using two BMD approaches: (i) an in-house CLSI/EUCAST-compliant BMD^4,5^ using Chelex-prepared ID-CAMHB and (ii) the commercial UMIC BMD system. The in-house BMD was considered the reference method for comparison of susceptibility categorization and MIC distributions. Fresh cefiderocol stock solutions were prepared and tested over a concentration range of 0.03–32 mg/L and a final inoculum of 5 × 10^5^ CFU/mL. For the UMIC system, manufacturer-provided ID-CAMHB and predefined cefiderocol concentrations (0.03–32 mg/L) were used with a final inoculum of approximately 7.5 × 10^5^ CFU/mL. MICs were interpreted according to EUCAST breakpoints, with isolates classified as susceptible (≤2 mg/L) or resistant (>2 mg/L).^9^

### ICP-MS determination of cation concentrations

Calcium, magnesium, zinc and iron concentrations of the in-house ID-CAMHB and the commercial UMIC medium were determined by inductively coupled plasma mass spectrometry (ICP-MS) at Indikator GmbH (Wuppertal, Germany). ICP-MS confirmed calcium, magnesium, zinc and iron concentrations of 21, 10, 0.53 and <0.1 mg/L, respectively, in the in-house medium, which were within the CLSI/EUCAST-recommended ranges (Ca 20–25 mg/L, Mg 10–12.5 mg/L and Zn 0.5–1.0 mg/L).^4,5^ In contrast, corresponding concentrations of 8, 3, 0.33 and <0.1 mg/L were measured in the commercial UMIC medium. All commercial UMIC media used in this study originated from the same production batch (lot). Cation concentrations were determined using material obtained from a single package of this batch.

### Quality control strains

*E. coli* ATCC 25922 and *Pseudomonas aeruginosa* ATCC 27853 were used for QC.

### Statistical analysis

Essential agreement and bias were assessed according to ISO 20776-2:2021.^10^ Categorical agreement, categorical discrepancies and MIC distributions were evaluated for the two BMD methods according to EUCAST breakpoints.^9^

## Results and discussion

### QC results

Both BMD methods were evaluated using QC strains. For the in-house BMD, MICs of 0.5 mg/L were obtained for *E. coli* ATCC 25922 and *P. aeruginosa* ATCC 27853, whereas the UMIC system yielded MICs of 0.25 and 0.5 mg/L, respectively. For both BMD methods, observed MICs were within the cefiderocol QC range (0.06–0.5 mg/L) established in the CLSI M23-A4 Tier 2 multicentre study, with one result matching the modal MIC value of 0.25 mg/L.^11,12^ The multicentre study established these QC ranges despite variability in MIC values associated with the use of ID-CAMHB prepared from CAMHB supplied by different manufacturers. Thus, acceptable QC performance alone does not indicate identical testing conditions or equivalent MIC values.^7^ In addition, the mean inhibition zone diameters of 27 mm for *E. coli* ATCC 25922 and 26 mm for *P. aeruginosa* ATCC 27853 matched the EUCAST target values for cefiderocol (24–30 mm for *E. coli* and 23–29 mm for *P. aeruginosa*) and were within the established QC ranges.^12^

### DDT versus MIC comparison

**Figure 1.**
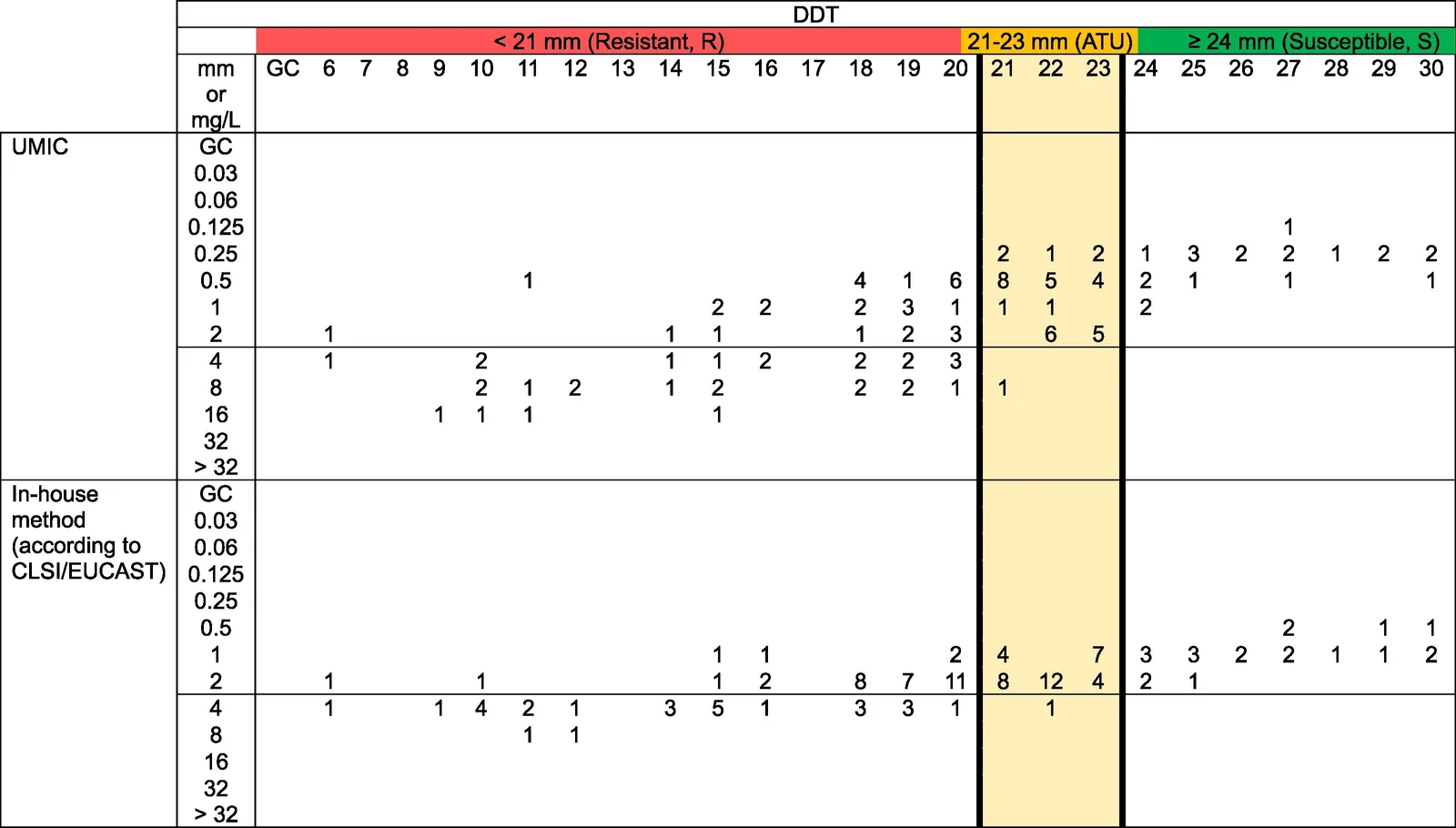
Comparison of DDT results with UMIC BMD and in-house BMD results for 119 *E. coli* and *K. pneumoniae* isolates. DDT results are shown in mm, while MICs were determined in mg/L. According to EUCAST breakpoints, DDT results are interpreted as follows: <21 mm resistant (R), 21–23 mm within the EUCAST ATU and ≥24 mm susceptible (S). MIC breakpoints are ≤2 mg/L susceptible (S) and >2 mg/L resistant (R).

Comparison of DDT results with MIC-based susceptibility categorization revealed substantial variability, particularly among isolates with reduced inhibition zone diameters (Figure 1). Within the DDT-resistant category (<21 mm), in-house reference BMD classified 35 isolates as susceptible and 27 as resistant, whereas UMIC BMD classified 31 isolates as susceptible and 31 as resistant. Several isolates were categorized differently by UMIC than by the in-house reference method, indicating limited interchangeability. Agreement of the UMIC BMD with the in-house reference BMD method was considerably higher for isolates within the EUCAST ATU. Within the ATU, both the in-house reference BMD method and the UMIC BMD classified one isolate as resistant; however, these represented different isolates. All remaining isolates within the ATU were categorized as susceptible by both methods. Likewise, all isolates with inhibition zone diameters of 24–30 mm were categorized as susceptible by both BMD methods, demonstrating excellent agreement for clearly susceptible isolates.

### MIC distribution differences

**Figure 2.**
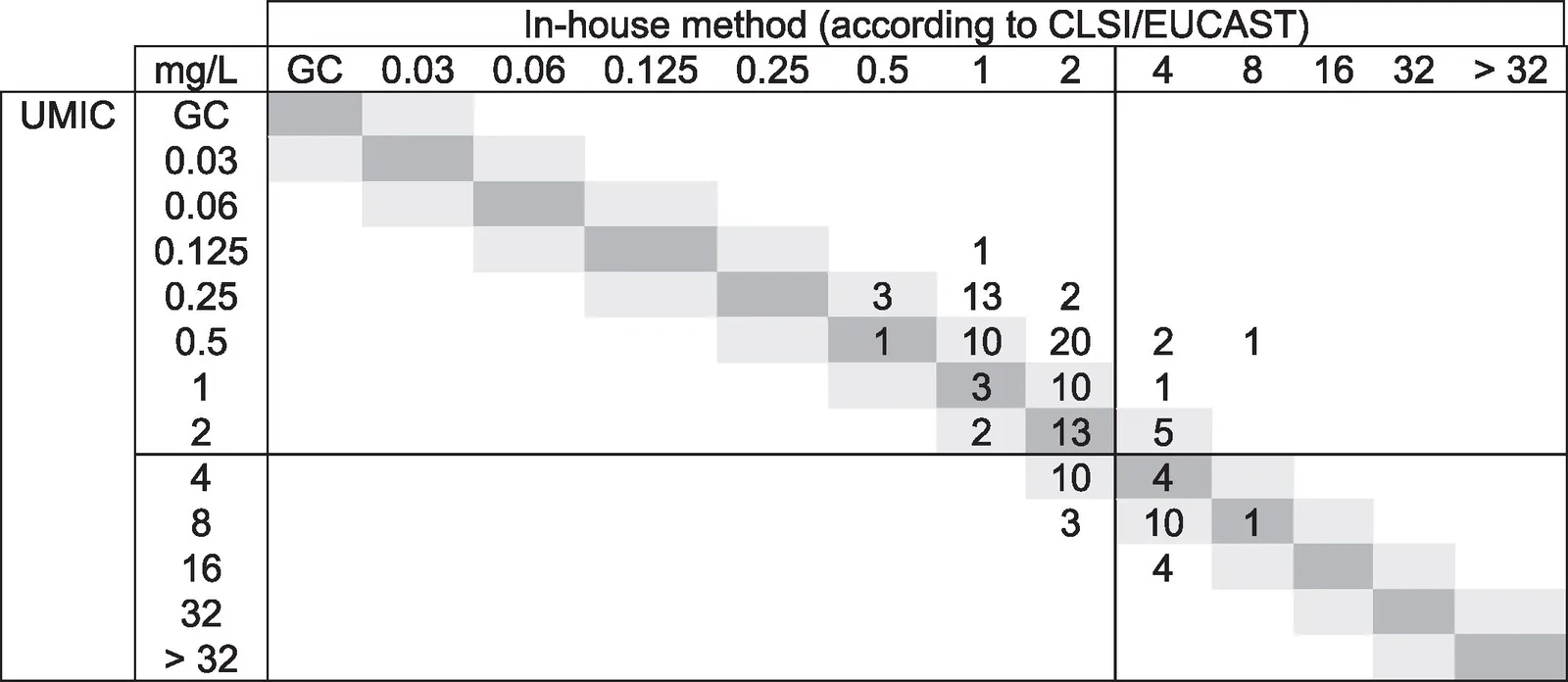
Comparison of cefiderocol MICs determined by UMIC BMD and in-house BMD for 119 *E. coli* and *K. pneumoniae* isolates. The diagonal line indicates perfect agreement between the two methods. MIC breakpoints according to EUCAST are ≤2 mg/L susceptible (S) and >2 mg/L resistant (R), highlighting concordance or discrepancies between UMIC BMD and in-house BMD results.

The two methods generated distinct MIC distribution patterns (Figure 2). In-house BMD produced marked clustering at MICs of 1, 2 and 4 mg/L across the complete range of DDT inhibition zone diameters (6–30 mm), with 113 of 119 isolates (95%) yielding MICs within this range, whereas MICs obtained with UMIC were distributed across a substantially broader range of dilution steps. Consistent with these differences, categorical agreement between in-house BMD and UMIC BMD was 81.5% (97/119 isolates), whereas 18.5% (22/119 isolates) showed discrepant susceptibility classifications. Most discrepant susceptibility classifications occurred at MICs within one or two 2-fold dilution steps of the EUCAST breakpoint.^9^ Essential agreement (±1 log_2_ dilution) was only 60.5% (72/119 isolates), indicating limited agreement at the MIC level. The relatively low essential agreement is likely attributable to the systematic differences in MIC distributions observed between the two methods. The deliberate inclusion of isolates covering a broad susceptibility range, including 36 isolates within the EUCAST ATU, may also have contributed to the observed agreement estimates. Consistent with these findings, bias analysis according to ISO 20776–2:2021 demonstrated a negative bias of −32.8% for the UMIC system relative to the in-house reference BMD, indicating a systematic tendency towards lower MIC values.^10^ The in-house CLSI/EUCAST-compliant BMD served as the reference method; however, the observed discrepancies indicate that the two test systems are not fully interchangeable. Further clinical outcome data are needed to determine the therapeutic relevance of these methodological differences.

### Methodological considerations

The observed MIC distribution differences may be related to methodological differences between the two BMD systems. Although the in-house CLSI/EUCAST-compliant BMD consistently clustered MICs at 1, 2 and 4 mg/L across the full range of inhibition zone diameters, the UMIC system yielded a substantially broader MIC distribution. These findings are unlikely to be explained solely by biological differences between isolates. The in-house CLSI/EUCAST-compliant assay used Chelex-prepared ID-CAMHB with recommended cation concentrations.^4,5^ In contrast, the UMIC system differed in several methodological aspects, including medium composition, inoculum density and antibiotic preparation. The UMIC medium contained substantially lower calcium, magnesium and zinc concentrations than recommended by CLSI and EUCAST guidance.^4,5^ However, the individual contribution of each methodological factor could not be determined. The pronounced clustering observed with the in-house assay may reduce the resolution of MIC measurements around the clinical breakpoint, whereas the broader UMIC MIC distribution may increase variability in susceptibility categorization. These methodological differences plausibly explain the distinct MIC distributions and low essential agreement. Because cefiderocol susceptibility interpretation is based on a single clinical breakpoint, even small MIC differences may influence susceptibility categorization and therapeutic decision-making.^6,9^

### Reproducibility/literature comparison

Consistent with our findings, previous studies demonstrated that ID-CAMHB prepared from CAMHB supplied by different manufacturers can produce systematic cefiderocol MIC shifts despite good within-medium reproducibility.^7,13^ Categorical discrepancies between DDT and a commercial cefiderocol BMD method have also been reported in a prospective multicentre evaluation, despite high overall agreement.^8^ These findings support the use of the CLSI/EUCAST-compliant reference BMD when evaluating commercial cefiderocol susceptibility testing systems. A limitation of this study is that isolates were selected to cover a broad range of cefiderocol inhibition zone diameters rather than representing a consecutive clinical collection. While this approach enabled assessment of method performance across the full susceptibility spectrum, it may have influenced agreement estimates and therefore does not necessarily reflect routine clinical practice. A further limitation is that intra-laboratory reproducibility of cefiderocol MIC testing was not systematically evaluated. Therefore, reproducibility was inferred from published studies rather than assessed experimentally.^7,13^

## Conclusion

These findings indicate that acceptable QC performance alone does not ensure methodological equivalence of cefiderocol MIC results. Differences in medium composition and other methodological factors may have contributed to the observed MIC distributions, particularly near the EUCAST breakpoints. The present study extends previous observations by directly comparing the commercial UMIC system with a CLSI/EUCAST-compliant reference BMD, highlighting systematic differences in MIC distributions between the two methods.
